# 

**Published:** 1885-05

**Authors:** 


### CONTENTS.

PROCEEDINGS.

Forty-first Annual Meeting of the Mississippi Valley Association of Dental Surgeons.......... 213

COMMUNICATIONS.

A Protest against Quackery.......... 234
The Inadequacy of our Resources in Operative Dentistry.......... 238
Textile Foil.......... 245

SELECTIONS.

The Removal of Stains from the Teeth, etc.,.......... 249
Dyspepsia and Indigestion.......... 252
Infant Feeding.......... 254
The Application of Dentistry to the Detection of Crime.......... 254
Choice of Teeth for Extraction for the Purpose of Uniformity.......... 255
Japanese Teeth.......... 255
Stretching of the Lingual Nerve for Neuralgia.......... 256
Responsibility for Sickness.......... 256
SALMAGUNDI.......... 257

EDITORIAL.

The Societies' International Medical Congress.......... 260
Northern Ohio Dental Association.......... 264
Mad River Dental Society.......... 264

